# Swimming through asymmetry: zebrafish as a model for brain and behavior lateralization

**DOI:** 10.3389/fnbeh.2025.1527572

**Published:** 2025-01-20

**Authors:** Alessandra Gobbo, Andrea Messina, Giorgio Vallortigara

**Affiliations:** Centre for Mind/Brain Sciences, University of Trento, Rovereto, Italy

**Keywords:** behavioral lateralization, brain asymmetry, genetics, *Danio rerio*, neurodevelomental disorders

## Abstract

The left and right sides of the brain show anatomical, neurochemical and functional differences. In the past century, brain and behavior lateralization was considered a human peculiarity associated with language and handedness. However, nowadays lateralization is known to occur among all vertebrates, from primates to fish. Fish, especially zebrafish (*Danio rerio*), have emerged as a crucial model for exploring the evolution and mechanisms of brain asymmetry. This review summarizes recent advances in zebrafish research on brain lateralization, highlighting how genetic tools, imaging, and transgenic methods have been used to investigate left–right asymmetries and their impact on sensory, cognitive, and social behaviors including possible links to neurodevelopmental and neurodegenerative disorders.

## 1 Introduction

The term “brain lateralization” refers to the different specializations of the left and right side of the nervous system. For more than one century, lateralization was considered a uniquely human characteristic associated with handedness and language (McManus, 1999; Mac Neilage et al., 2009). However, early evidence acquired in the 1970s challenged this view proving that lateralization was present in non-human species (Nottebohm, 1971; Denenberg et al., 1978; Rogers and Anson, 1979). Since then, studies showed a consistent pattern of lateralization across animals, with the left side of the brain primarily involved in the categorization of stimuli and focusing attention, and the right side specialized in emotional and social processes, as well as in reacting to new and unexpected stimuli (Vallortigara et al., 2011; Frasnelli et al., 2012; Ströckens et al., 2013; Ocklenburg et al., 2013; Rogers et al., 2013; Rogers and Vallortigara, 2017; Vallortigara and Rogers, 2020; Vallortigara and Versace, 2017; Mac Neilage et al., 2009).

In the last 20 years, studies on fish have also contributed significantly to this field (Vallortigara and Bisazza, 2002; Bisazza and Brown, 2011; Duboc et al., 2015), and zebrafish (*Danio rerio*) has become a model for studying asymmetries of the vertebrate brain (Roussigne et al., 2012). Specifically, with their laterally positioned eyes with limited overlap and almost completely crossed optic chiasm zebrafish provide an excellent model for studying eye preferences (e.g., Sovrano et al., 2001; also reviewed in Stancher et al., 2018). Furthermore, the asymmetric development of the zebrafish epithalamus associated with the expression of specific lateralized neural markers offers the opportunity to examine the relationship between anatomical and functional asymmetries (Concha and Wilson, 2001; Andrew et al., 2009; Beretta et al., 2012; Agostini et al., 2022).

In this mini-review, we will describe the developmental and molecular processes involved in the building of brain asymmetries in zebrafish, even in relation to neurodevelopmental diseases associated with altered brain lateralization.

## 2 Behavioral asymmetries in zebrafish

**Figure 1:**
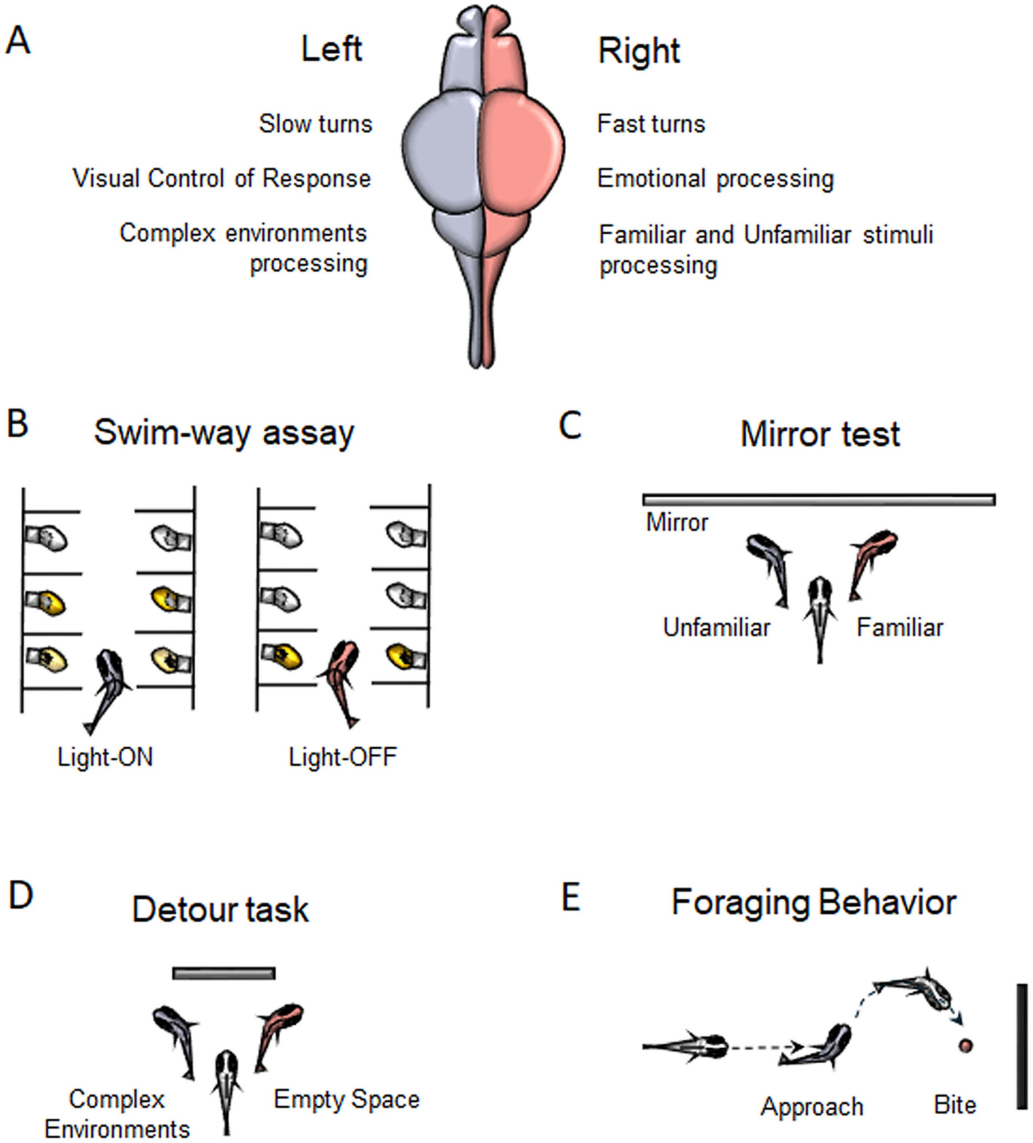
Behavioral Asymmetries in zebrafish. **(A)** Main functions described for the left and right hemisphere in zebrafish. **(B)** Motor turning biases associated to Swim-way assay. **(C)** Preference for left-or right-eye viewing for familiar/unfamiliar stimuli during Mirror test. **(D)** Lateralized behavior to swim around a barrier in the Detour task. **(E)** Foraging behavior showing a right-eye viewing associated to bite.

Evidence of motor and sensory asymmetries (Figure 1A) in fish is well documented (Miletto Petrazzini et al., 2020). One of the earliest examples is associated with the C-start escape response. This behavior involves a unilateral muscle contraction, coordinated by the Mauthner cells and reticulospinal neurons of the hindbrain, followed by a tip-over of the tail that allows the fish to escape quickly (Kohashi and Oda, 2008; Stancher et al., 2018). Heuts (1999) observed that zebrafish exhibit a rightward bias in fast turns and a leftward bias in slow turns, likely due to neural and muscular asymmetry: fast swimming relies on white muscles, and slow swimming engages red muscles, each asymmetrically distributed on either side of the body. Although there is one report that this behavior is not lateralized in zebrafish (Satou et al., 2009), several studies have found evidence of lateralized C-start responses in other teleost species, such as *Cymatogaster aggregate* (Dadda et al., 2010b), *Jenynsia lineata* (Bisazza et al., 1997a; Bisazza et al., 1997b), and *Girardinus falcatus* (Cantalupo et al., 1995).

Another method used to assess motor biases in zebrafish is the swim-way assay (Figure 1B), which consists of multiple individually lit chambers connected by small corridors through which larvae can swim (Burgess and Granato, 2007). Zebrafish larvae are generally attracted to light and tend to avoid darkness and, in response to abrupt lights-off events, they display characteristic locomotor responses (Burgess and Granato, 2007). In the swim-way test, the larva is placed in the first illuminated compartment, then the light is gradually switched off, while the light in the second compartment is turned on, prompting the larva to move forward. This process is repeated until the larva either turns into a specific compartment or reaches the last chamber of the apparatus without turning. The larvae exhibited lateralized turning behaviors: under light conditions, they showed a strong tendency to turn left, whereas in darkness following abrupt light-off events, they showed a significant preference for turning right (Watkins et al., 2004). This rightward bias, likely associated with the startle response, can be attributed to the directional bias of the Mauthner cells, which are influenced by the layout of environmental obstacles, thereby facilitating rapid escape. Additionally, the preferential use of the right eye to monitor the escape route may contribute to initiating locomotion with a rightward bias (Watkins et al., 2004).

Most fish research has focused on eye preferences, with evidence suggesting that the left eye (right hemisphere) processes differences between familiar and unfamiliar objects, while the right eye (left hemisphere) is involved in guiding and regulating action-oriented behaviors based on visual input such as approaching prey, avoiding predators, or navigating around obstacles (Vallortigara, 1992; Rogers et al., 2013). The mirror test (Figure 1C) is commonly used to assess visual lateralization, with zebrafish larvae showing a strong preference for left-eye viewing of their reflection. This left bias decreases around 14 days and increases by 21 days, influenced by factors like age and genetics (Sovrano and Andrew, 2006). Adult zebrafish also favor the left eye when viewing their reflection or groups of conspecifics (Sovrano et al., 1999). Treating larvae with valproic acid (VPA), which impacts social abilities and induces autism spectrum disorder (ASD) traits, disrupts this bias, resulting in no preference for eye use during the test (Messina et al., 2024). Additionally, zebrafish show a left-eye bias when inspecting familiar patterns but not unfamiliar ones. Even without prior exposure to their reflection, previously encountered visual patterns engage the left eye, suggesting right hemisphere dominance for familiarity (Rogers et al., 2013; Sovrano, 2004).

Abnormal behaviors during the mirror test have been observed in Tg(*foxD3*:GFP) zebrafish larvae and adults injected with *southpaw* antisense morpholino. These fish express Green Fluorescent Protein (GFP) under the *foxd3* promoter, marking pineal and parapineal precursors during development. Morpholino injections result in reversed epithalamic asymmetry. Right-sided parapineal (Rpp) larvae show delayed swimming and reduced exploration during the mirror test, suggesting heightened fear and anxiety (Facchin et al., 2009; Facchin et al., 2015). In the novel tank test, adult Rpp zebrafish exhibit more time at the bottom of the tank, a measure of anxiety, compared to left-sided parapineal (Lpp) controls (Facchin et al., 2015). Similarly, in the confined box test, Rpp adults delay exiting when exposed to a bright tank, indicating elevated levels of fear (Facchin et al., 2015). These altered behaviors are attributable to elevated cortisol levels, and thus high anxiety levels, found in Rpp subjects, compared to Lpp controls, that indeed can be restored by anxiolytic treatment (Facchin et al., 2015).

Zebrafish exhibit opposite eye-use preferences. They rely on the left eye for routine behavioral control and social inspections of familiar species, while the right eye is used for detecting potential threats, analyzing complex environments, and responding to potentially dangerous species (Miklosi et al., 1997; Vallortigara and Rogers, 2005; Rogers et al., 2013). This lateralization is also evident in detour tests (Figure 1D), where zebrafish swimming around a barrier prefer to view empty spaces with the left eye and analyze intricate surroundings with the right (Miklosi et al., 1997).

Asymmetrical biases have been found in foraging behaviors as well (Figure 1E). When approaching a target to bite, zebrafish tend to favor the right eye and to approach from the left side. Miklosi and Andrew (1999) reported that when zebrafish are presented with a novel object associated with food, they initially exhibit right-eye use and biting behavior, both of which decrease over subsequent trials.

## 3 Brain asymmetries: habenular complex and dorsal pallium

**Figure 2:**
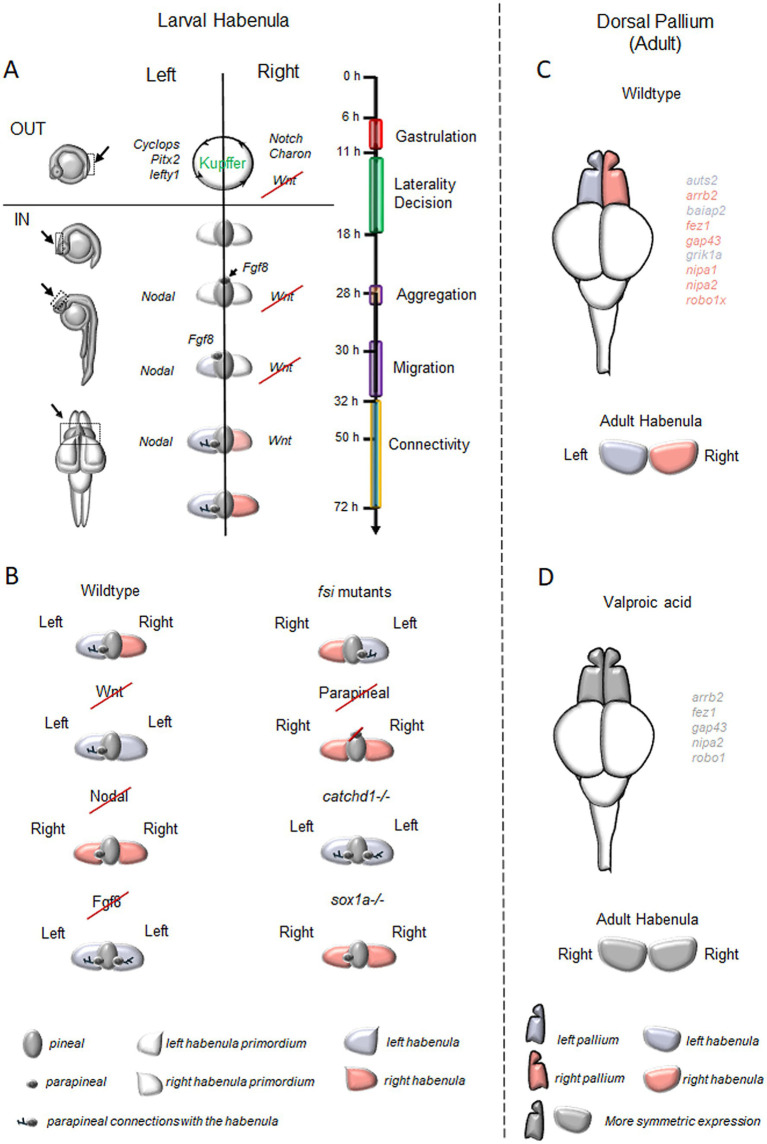
Brain Asymmetries in the zebrafish forebrain. **(A)** Timeline of key processes involved in the generation of neuroanatomical asymmetry in the zebrafish habenular complex and the regulatory genes of epithalamic asymmetry. In brief, between 11-and 18-h post-fertilization (hpf), Notch signaling influences the directional fluid flow generated by the ciliated cells of Kupffer’s vesicle, determining the positioning of Nodal-related genes on the left side of the zebrafish embryos (Raya et al., 2003) and Nodal inhibitors along with WNT signaling molecules on the right (Hashimoto et al., 2004; Hojo et al., 2007). By 28 hpf, FGF8 disrupts the symmetry of epithalamic structures (Neugebauer and Yost, 2014) and, in coordination with Nodal, establishes epithalamic asymmetry. This process supports the migration of parapineal cells to the embryo’s left side (30–32 hpf; Concha et al., 2000; Long et al., 2003; Carl et al., 2007; Inbal et al., 2007; Snelson and Gamse, 2009; Roussigne et al., 2012; Duboc et al., 2015) and forms the parapineal connections to the left habenular nuclei. Additionally, FGF8 activates Wnt signaling on the right side, contributing to the specification of the right habenular nuclei between 50 and 72 hpf (Carl et al., 2007; Hüsken and Carl, 2013). **(B)** Typical and atypical patterns of brain asymmetry described in studies on zebrafish habenular functions. **(C)** Asymmetric distribution of genes linked to neurodevelopmental disorders in the zebrafish dorsal pallium. **(D)** Effects of Valproic Acid (VPA) exposure on asymmetrically expressed neurodevelopmental disorder-associated genes in the dorsal pallium and habenula. In C and D, genes labeled in violet are predominantly expressed on the left side in adult zebrafish, while pink indicates right-side dominance. VPA treatment disrupts these patterns, leading to symmetric expression (labeled in gray).

The epithalamus (Figure 2A) is a structure that displays left–right differences across vertebrates, including zebrafish (Concha and Wilson, 2001; Duboué et al., 2017). The epithalamus includes the habenula, the pineal complex, and the stria medullaris, a bundle of fibers connecting to the habenula (Beretta et al., 2012; Bianco and Wilson, 2009; Aizawa et al., 2011). The pineal complex includes the pineal gland (or epiphysis) and the parapineal organ that shows an asymmetric position (Concha et al., 2000). While the pineal organ does not establish symmetrical or asymmetrical connections with the habenula, the parapineal organ is located on the left side of the pineal gland and projects solely to the left dorsal lateral subnucleus of the habenula (Concha et al., 2000; Gamse et al., 2005).

The habenula is the most asymmetric region of the epithalamus and plays the role of a critical relay station connecting the forebrain to the brain stem in all vertebrate taxa (Aizawa, 2013). In zebrafish, the habenula is subdivided into two different compartments rotated 90 degrees counterclockwise compared to mammalian ones (Amo et al., 2010; Güntürkün and Ocklenburg, 2017). The dorsal nucleus of the zebrafish habenula corresponds to the mammalian medial nucleus and releases acetylcholine and the neuropeptides Substance P, while the ventral nucleus is homologous to the lateral habenula of mammals and contains glutamatergic neurons (Gamse et al., 2002; Agetsuma et al., 2010; Facchin et al., 2015). Similarly to other vertebrates (Miyasaka et al., 2009; Roussigne et al., 2012; Bühler and Carl, 2021), the habenular nuclei of zebrafish exhibit asymmetrical efferent connections to the interpeduncular nucleus (IPN) of the midbrain (Gamse et al., 2005; Bianco et al., 2008).

Additional forebrain asymmetries have been also identified regarding the distribution of a set of genes linked to neurodevelopmental disorders in the zebrafish dorsal pallium (Figure 2C), with some autism-related genes showing leftward (e.g., *auts2*, *baiap2*) and others rightward asymmetry (e.g., *arrb2*, *fez1*, *gap43*, *robo1*). Furthermore, genes associated with developmental dyscalculia also show lateralization, with some leftward (e.g., *grik1a*) and others rightward (e.g., *nipa1*, *nipa2*, *robo1*; Messina et al., 2021). Similar differential gene expression between the left and right hemispheres has been reported even in mammals, including humans (Sun et al., 2005; Ribasés et al., 2009; Samara et al., 2011; Hüsken and Carl, 2013).

## 4 Genes regulating epithalamic asymmetry

In zebrafish, the epithalamus initially develops as a bilaterally symmetrical structure with dorsal and ventral domains, where four signaling pathways work to establish asymmetry: Nodal, Fibroblast Growth Factors (FGFs), Notch, and Wnt/*β*-catenin (Figure 2A; Concha et al., 2003).

The Nodal pathway disrupts symmetry through signals from the dorsal and lateral mesoderm, with Nodal-related genes *cyclops* and *southpaw* activating transcription factors (e.g., *Otx5, Noto, Foxd3*) critical for parapineal organ development on the left side of the pineal complex (Erter et al., 1998; Sampath et al., 1998; Rebagliati et al., 1998). Experiments with mutant zebrafish lacking the mesoderm of the notochord and in which *cyclops* was bilaterally expressed in the developing tissues of the dorsal diencephalon highlighted the importance of dorsal mesoderm signals in maintaining epithalamic asymmetry (Rebagliati et al., 1998; Bisgrove et al., 2000; Liang et al., 2000). Liang and colleagues further demonstrated that mesodermal signals influence the left-sided positioning of the pineal complex, through genes like *cyclops*, *antivin*, and *pitx2*, indicating that visceral laterality pathways could impact forebrain asymmetry (Roussigné et al., 2009).

Further evidence of the involvement of the Nodal pathway in the specification of epithalamic asymmetry emerged from studies on early habenular development. Investigating the habenular progenitor marker *cxcr4b* expressed in the parapineal cells before their migration, Roussigné et al. (2009) showed that the disruption of Nodal signaling resulted in the generation of symmetric habenular nuclei, indicating that this pathway not only governs laterality development but also contributes to the establishment of the brain asymmetry (Long et al., 2003). A similar result was obtained using the Nodal chemical inhibitor SB431542 or knocking-down *southpaw* leading to symmetric or mildly asymmetric structures that caused the downregulation of left-sided genes (such as *cyclops*, *pitx2*, *lefty1*, and *lefty2*) in the dorsal epithalamus and a failing of the lateralization process in the pineal complex (Long et al., 2003; Barth et al., 2005; Carl et al., 2007).

While Nodal signaling is essential for establishing forebrain asymmetry, Fibroblast Growth Factor (FGF) signaling serves as the initial trigger. Regan et al. (2009) showed that FGF8 is responsible for guiding the leftward migration of the parapineal complex. Zebrafish mutants lacking FGF8 or treated with FGF8 morpholinos failed to develop any asymmetry in the epithalamus and habenula due to the impaired migration toward the left side of parapineal cells, resulting in a symmetrical structure (Reifers et al., 1998; Draper et al., 2001; Neugebauer and Yost, 2014). Similarly, treatment with the FGF inhibitor SU5402 induced symmetry, but the defect was rescued with FGF8-soaked beads (Reifers et al., 1998). Neugebauer and Yost (2014) demonstrated that FGF signaling regulates the transcription factors *six3b* and *six7*, which specifically repress the Nodal target *lefty1*. Knockdown of *six3b* and *six7* led to a bilateral *lefty1* expression, while their overexpression suppressed *lefty1* on both sides (Melby et al., 1996). Furthermore, FGF signaling is crucial for midline organization and interacts with the Nodal pathway to maintain brain asymmetry (Inbal et al., 2007) and determining its directionality (Güntürkün and Ocklenburg, 2017).

During gastrulation, the Notch pathway is essential for establishing Nodal-mediated left–right asymmetry, particularly through the regulation of cilia length in the Kupffer’s Vesicle (Essner et al., 2017; Takeuchi et al., 2010; Hashimoto et al., 2004). These cilia generate a leftward fluid flow that directs Nodal signaling to the left side of the embryo and places the Nodal antagonist Charon to the right (Raya et al., 2003; Gourronc et al., 2007; Hojo et al., 2007). Bilateral microinjection of *Notch* mRNA caused overexpression of *ndr2/cyclops* and *pitx2*, genes typically confined to the left side of the embryo, which began to be expressed on both sides. This finding establishes a connection between Notch and Nodal signaling in regulating epithalamic asymmetry in fish (Carl et al., 2002).

Another important pathway that impacts brain asymmetry is the Wnt/*β*-catenin pathway, which acts upstream of Nodal at three stages: late gastrulation, somitogenesis, and epithalamic development (Concha et al., 2003; Inbal et al., 2007). For example, mutations in *axin/masterblind* (a Wnt inhibitor) or the Wnt inhibition via lithium chloride disrupted asymmetric Nodal gene distribution in the brain, while asymmetry of the lateral mesoderm was unaffected (Concha et al., 2003; Lagutin et al., 2003; Sagasti, 2007; Snelson and Gamse, 2009; Caron et al., 2012). Wnt signaling seems to contribute to Kupffer’s Vesicle development by activating the transcription factor *foxj1a* and reinforcing the role of Notch signaling in the positioning of Nodal-related genes on the left side of the forebrain (Concha et al., 2003; Hüsken et al., 2014). Finally, Wnt also regulates the transcription of *tcf7l2,* a factor that controls the acquisition of left–right dorsal habenular phenotype in the developing epithalamus of zebrafish (Guglielmi et al., 2020).

## 5 Tools to probe asymmetries in zebrafish

Zebrafish have played a key role in advancing our understanding of brain asymmetry and its development in vertebrates. Experimental protocols to genetically, chemically, environmentally, and surgically manipulate brain asymmetries in this species are available (Figure 2B).

The use of chemical drugs to block specific signaling pathways has contributed significantly to the understanding of the molecular mechanisms underlying the generation of the zebrafish epithalamic asymmetry. For instance, IWR-1 (an inhibitor of the Wnt pathway), stabilizing *axin* and contributing to the degradation of *beta-catenin*, disrupts Wnt signaling leading to a “double-left” habenula phenotype (Long et al., 2003; Andrew et al., 2009). On the contrary, SB431542 (a TGF-beta inhibitor) inhibits Nodal-related factors, resulting in “double-right” symmetric habenular structures (Andrew et al., 2009; Dreosti et al., 2014). Moreover, SU5402, an FGF receptor inhibitor, disrupts parapineal cell migration and induces symmetry in habenula acting on *lefty1* expression (Reifers et al., 1998).

Environmental conditions also affect brain asymmetry. Zebrafish and avian embryos raised in darkness or at lower temperatures during gastrulation show disrupted lateralization in habenula orientation (Inbal et al., 2007; Regan et al., 2009; Andrew et al., 2009; Keller and Ahrens, 2015; Rogers et al., 2013; Bühler and Carl, 2021; Versace et al., 2022; Costalunga et al., 2024), and a loss of lateralization in the ability to respond to visual and olfactory stimuli (Güntürkün and Kesch, 1987; Güntürkün et al., 2000; Dreosti et al., 2014).

Another common tool to probe the contribution of the parapineal cells to the development of habenular lateralization is two-photon laser microscopy ablation. This approach results in an increased proliferation of dorso-medial habenular neurons in the left hemisphere leading to embryos with a “double-right” phenotype, where the left dorsal habenula fails to develop its typical features (e.g., larger size, expanded dense neuropil, and increased *lov* expression) and mimics the right side, with a disruption of the usual asymmetry (Erter et al., 1998; Gamse et al., 2003; Gamse et al., 2005; Bianco and Wilson, 2009).

In recent years, the establishment of new optical tools and the generation of fluorescent sensors enhanced our possibility to track neural development with high spatial and temporal resolution (Keller and Ahrens, 2015). With these approaches, many genes involved in brain asymmetry, such as the potassium channel tetramerization domain-containing genes (*kctd12.1*, *kctd12.2*, and *kctd8*), have been identified and used as markers to map the habenula and its connections with other regions of the brain (Aizawa et al., 2005; Gamse et al., 2005; de Carvalho et al., 2014; Wang et al., 2021) or specific compartments of the habenulae (Kuan et al., 2007; Deguchi et al., 2009; Pandey et al., 2018; Carl et al., 2007; Agetsuma et al., 2010).

As previously mentioned, transgenic lines expressing GFP under the control of tissue-specific promoters can be useful tools to study epithalamic asymmetry in zebrafish. For example, the Tg(*foxD3*:GFP) has been extensively utilized to monitor parapineal development and positioning (Snelson et al., 2008; see also Miletto Petrazzini et al., 2020). Studying the connections between the telencephalic nuclei and interpeduncular nucleus of the midbrain in the Tg(*foxD3*:GFP) transgenic line, Aizawa et al. (2005) revealed a mechanism for bilateral information transfer in the brain, preserving left–right coding crucial for functional lateralization. Gamse et al. (2005) reported that the parapineal laser-ablation disrupts habenular asymmetry altering the dorsoventral distribution of habenular innervations. The habenular innervations require parapineal instructions but are also supported by additional developmental mechanisms contributing to the lateralization of these circuits (Bianco et al., 2008). Moreover, the Tg(*phlx2a*:GFP) line revealed bulbo-habenular projections from the olfactory bulb to the right habenula (Miyasaka et al., 2009). In summary, zebrafish GFP transgenic-lines have advanced studies on visual and motor laterality (Dadda et al., 2010a), lateralized habenular nuclei responses to stimuli (Krishnan et al., 2014; Dreosti et al., 2014), and the role played by epithalamic asymmetry in anxiety (Facchin et al., 2015) and in fear response (Duboué et al., 2017).

Finally, CRISPR/Cas9 technology has been used to generate knockout lines for asymmetry studies. For example, the *sox1a* mutant line presents a “double-right” habenular phenotype, demonstrating the importance of this gene in establishing brain asymmetry (Lekk et al., 2019). On the other hand, the *cachd1* mutant line resulted in a symmetric “double-left” habenula supporting the role of Wnt signaling in the establishment of the epithalamic asymmetry (Powell et al., 2024).

## 6 Brain asymmetry and human disorders

It is well known that the left and right hemispheres of the human brain exhibit anatomical and functional asymmetries (Rogers et al., 2013). In humans, brain asymmetries are first observed at around 29–31 weeks of gestation, and they continue to develop into childhood and adulthood (Toga and Thompson, 2003). These asymmetries are linked to differences in maturation rates, dendritic branching, metabolism, and functions between the two hemispheres of the brain. The specific pattern of asymmetry varies depending on factors such as handedness, gender, age, and genetic and hormonal influences (Toga and Thompson, 2003; Güntürkün et al., 2020).

Functional and structural lateralization are important for cognitive development (Toga and Thompson, 2003). Altered lateralization is linked to reduced cognitive abilities and neuropsychiatric disorders (Forrester and Todd, 2018), including dyslexia, reading disorders, and right-hemisphere speech dominance (Hynd et al., 1990). Neurodegenerative diseases like semantic dementia and Alzheimer’s exhibit left hemisphere vulnerability with asymmetric atrophy (Thompson et al., 2003). Severe left hemisphere dysfunction also leads to developmental dyscalculia, especially in complex arithmetic (Shalev et al., 1995). Zebrafish models for these disorders offer valuable tools to explore cerebral lateralization and related conditions such as dyslexia, dementia, and dyscalculia (Gostic et al., 2019; Newman et al., 2014; Torres-Perez et al., 2023, 2024).

Alterations in cerebral lateralization are evident in Williams-Beuren syndrome (WBS), a genetically defined neurodevelopmental disorder in which changes in the pattern of cerebral lateralization may underlie its distinctive intellectual disability and neurocognitive profile, which includes marked anxiety, hypersociability, poor visuospatial skills, and developmental dyscalculia, with preserved language abilities (Pober, 2010; Bellugi et al., 2000; Mervis et al., 1999). Zebrafish lines for WBS genes reveal alterations in brain lateralization-dependent behaviors. Mutants for *baz1b* show social impairments (Torres-Perez et al., 2023), while *fzd9b* (Torres-Perez et al., 2024) and *rfc2* (Park et al., 2024) mutants exhibit altered anxiety. These findings highlight the potential use of zebrafish as a model to shed light on WBS-related brain lateralization.

Loss of cerebral lateralization is linked to autism (Forrester and Todd, 2018), a condition marked by atypical social interaction, communication, restricted interests, and sensory processing issues. Autism Spectrum Disorder (ASD) is associated with deficits in language processing, abnormal hemispheric activation to speech, and atypical handedness (Kjelgaard and Tager-Flusberg, 2001; Lombardo et al., 2015). Individuals with ASD lack a left visual field bias for face and emotion processing, reflecting altered lateralization (Dundas et al., 2012; Masulli et al., 2022). Changes in activation patterns in regions processing facial configurations have also been observed (McCleery et al., 2009; Keehn et al., 2015). Zebrafish provide a valuable model for examining neurodevelopmental and neurodegenerative disorders where lateralization anomalies (Figure 2D) are apparent but poorly understood, as we previously reported using valproic acid to mimic autism spectrum disorders and showing its impact on the ability to affect social visual lateralization and the asymmetric genes expression of typical habenular and pallial markers (Messina et al., 2024).

## 7 Ecology, ethology and evolution of lateralization

How could lateralized behaviors have evolved? At the individual level, a plausible explanation is that, in terms of survival of the organism, the benefits of lateralized responses outweigh the ecological disadvantages associated with a lateralized brain (Vallortigara, 2006). The potential advantages of lateral biases can be grouped into three main categories. First, lateralization increases neural efficiency by allowing the non-specialized hemisphere to remain available for other tasks (Denenberg, 1981). Second, it helps prevent the spontaneous initiation of conflicting responses in animals with laterally positioned eyes (Andrew, 1991; Cantalupo et al., 1995; Vallortigara, 2000). Third, it enhances the brain capacity for simultaneous and parallel processing (Rogers et al., 2004).

However, these benefits do not fully explain the alignment of asymmetries among populations. Lateralization at the population level can introduce challenges. Because the environment is symmetric, lateralized responses can leave an organism vulnerable to predators on one side or reduce its effectiveness in foraging or attacking prey (Corballis, 1997; Vallortigara and Rogers, 2005). Furthermore, when most individuals in a population share the same directional bias, their behavior becomes predictable, which may represent a disadvantage (Hori, 1993; Ghirlanda and Vallortigara, 2004).

To address how population-level lateralization arises despite these potential drawbacks, Ghirlanda and Vallortigara (2004) proposed that it may have evolved as an “evolutionarily stable strategy” (Maynard Smith, 1982) to coordinate behavior among asymmetric individuals. By applying a game-theoretical model to predator–prey interactions, they demonstrated that population-level lateralization can be evolutionarily stable, emerging when the fitness of an asymmetric organism depends on the actions of other asymmetric individuals (Ghirlanda and Vallortigara, 2004; Vallortigara and Rogers, 2005). These theoretical findings suggest that while individual-level lateralization provides functional advantages, population-level lateralization may be a cooperative adaptation driven by ecological and social pressures. This alignment facilitates coordination among individuals and highlights the intricate interplay between individual fitness and group dynamics in shaping evolutionary strategies. There has been also recent mathematical development of the theory that cannot be treated in this short review (but see Ghirlanda et al., 2009; Vallortigara and Vitiello, 2024).

## 8 Conclusion

Brain lateralization is widespread among vertebrates, but its genetical bases remain poorly understood. Zebrafish are a valuable animal model for studying brain asymmetry. In humans, atypical cerebral asymmetry is associated with neurodevelopmental disorders like autism, but ethical constraints limit research. On the other hand, zebrafish may facilitate the study of gene–environment interactions that influence lateralization and impact social, sensory, and cognitive behaviors. As research progresses, zebrafish may improve our understanding of lateralization’s evolutionary role and its relevance to neurological disorders, offering a promising avenue for research and therapies.

## References

[ref1] Agetsuma M. Aizawa H. Aoki T. Nakayama R. Takahoko M. Goto M.. (2010). The habenula is crucial for experience-dependent modification of fear responses in zebrafish. Nat. Neurosci. 13, 1354–1356. doi: 10.1038/nn.2654, PMID: 20935642

[ref2] Agostini C. Bühler A. Antico Calderone A. Aadepu N. Herder C. Loosli F.. (2022). Conserved and diverged asymmetric gene expression in the brain of teleosts. Front. Cell Develop. Biol. 10:1005776. doi: 10.3389/fcell.2022.1005776, PMID: 36211473 PMC9532764

[ref3] Aizawa H. (2013). Habenula and the asymmetric development of the vertebrate brain. Anat. Sci. Int. 88, 1–9. doi: 10.1007/s12565-012-0158-6, PMID: 23086722

[ref4] Aizawa H. Amo R. Okamoto H. (2011). Phylogeny and ontogeny of the habenular structure. Front. Neurosci. 5:138. doi: 10.3389/fnins.2011.00138, PMID: 22203792 PMC3244072

[ref5] Aizawa H. Bianco I. H. Hamaoka T. Miyashita T. Uemura O. Concha M. L.. (2005). Laterotopic representation of left-right information onto the dorso-ventral axis of a zebrafish midbrain target nucleus. Curr. Biol. 15, 238–243. doi: 10.1016/j.cub.2005.01.014, PMID: 15694307 PMC2790415

[ref7] Amo R. Aizawa H. Takahoko M. Kobayashi M. Takahashi R. Aoki T.. (2010). Identification of the zebrafish ventral habenula as a homolog of the mammalian lateral habenula. J. Neurosci. 30, 1566–1574. doi: 10.1523/JNEUROSCI.3690-09.2010, PMID: 20107084 PMC6633804

[ref8] Andrew R. J. (1991). Neural and Behavioural plasticity: The use of the domestic Chick as a model. Oxford: Oxford University Press.

[ref9] Andrew R. J. Osorio D. Budaev S. (2009). Light during embryonic development modulates patterns of lateralization strongly and similarly in both zebrafish and chick. Philos. Trans. Royal Society B: Biolog. Sci. 364, 983–989. doi: 10.1098/rstb.2008.0241, PMID: 19064353 PMC2666083

[ref11] Barth K. A. Miklosi A. Watkins J. Bianco I. H. Wilson S. W. Andrew R. J. (2005). Fsi zebrafish show concordant reversal of laterality of viscera, neuroanatomy, and a subset of behavioural responses. Curr. Biol. 15, 844–850. doi: 10.1016/j.cub.2005.03.047, PMID: 15886103 PMC2790416

[ref12] Bellugi U. Lichtenberger L. Jones W. Lai Z. (2000). I. The neurocognitive profile of Williams syndrome: a complex pattern of strengths and weaknesses. J. Cogn. Neurosci. 12, 7–29. doi: 10.1162/089892900561959, PMID: 10953231

[ref13] Beretta C. A. Dross N. Guiterrez-Triana J. A. Ryu S. Carl M. (2012). Habenula circuit development: past, present, and future. Front. Neurosci. 6:51. doi: 10.3389/fnins.2012.00051, PMID: 22536170 PMC3332237

[ref14] Bianco I. H. Carl M. Russell C. Clarke J. D. Wilson S. W. (2008). Brain asymmetry is encoded at the level of axon terminal morphology. Neural Dev. 3, 1–20. doi: 10.1186/1749-8104-3-9, PMID: 18377638 PMC2292717

[ref15] Bianco I. H. Wilson S. W. (2009). The habenular nuclei: a conserved asymmetric relay station in the vertebrate brain. Philos. Trans. Royal Society B: Biolog. Sci. 364, 1005–1020. doi: 10.1098/rstb.2008.0213, PMID: 19064356 PMC2666075

[ref16] Bisazza A. Brown C. (2011). “Lateralization of cognitive functions in fish” in Cognition and behavior. eds. Brown C. Laland K. Krause J. (Oxford: Wiley-Blackwell), 298–324.

[ref17] Bisazza A. Cantalupo C. Vallortigara G. (1997a). Lateral asymmetries during escape behavior in a species of teleost fish (*Jenynsia lineata*). Physiol. Behav. 61, 31–35. doi: 10.1016/S0031-9384(96)00308-3, PMID: 8976530

[ref18] Bisazza A. Pignatti R. Vallortigara G. (1997b). Laterality in detour behaviour: interspecific variation in poeciliid fish. Anim. Behav. 54, 1273–1281. doi: 10.1006/anbe.1997.0522, PMID: 9398380

[ref19] Bisgrove B. W. Essner J. J. Yost H. J. (2000). Multiple pathways in the midline regulate concordant brain, heart and gut left-right asymmetry. Development 127, 3567–3579. doi: 10.1242/dev.127.16.3567, PMID: 10903181

[ref20] Bühler A. Carl M. (2021). Zebrafish tools for deciphering habenular network-linked mental disorders. Biomol. Ther. 11:324. doi: 10.3390/biom11020324, PMID: 33672636 PMC7924194

[ref21] Burgess H. A. Granato M. (2007). Modulation of locomotor activity in larval zebrafish during light adaptation. J. Exp. Biol. 210, 2526–2539. doi: 10.1242/jeb.003939, PMID: 17601957

[ref22] Cantalupo C. Bisazza A. Vallortigara G. (1995). Lateralization of predator-evasion response in a teleost fish (*Girardinus falcatus*). Neuropsychologia 33, 1637–1646. doi: 10.1016/0028-3932(95)00043-7, PMID: 8745121

[ref23] Carl M. Bianco I. H. Bajoghli B. Aghaallaei N. Czerny T. Wilson S. W. (2007). Wnt/Axin 1/β-catenin signaling regulates asymmetric nodal activation, elaboration, and concordance of CNS asymmetries. Neuron 55, 393–405. doi: 10.1016/j.neuron.2007.07.007, PMID: 17678853 PMC1940036

[ref24] Carl M. Loosli F. Wittbrodt J. (2002). Six3 inactivation reveals its essential role for the formation and patterning of the vertebrate eye. Development. 129, 4057–4063. doi: 10.1242/dev.129.17.4057 12163408

[ref25] Caron A. Xu X. Lin X. (2012). Wnt/β-catenin signaling directly regulates Foxj1 expression and ciliogenesis in zebrafish Kupffer’s vesicle. Development 139, 514–524. doi: 10.1242/dev.071746, PMID: 22190638 PMC4074261

[ref26] Concha M. L. Burdine R. D. Russell C. Schier A. F. Wilson S. W. (2000). A nodal signaling pathway regulates the laterality of neuroanatomical asymmetries in the zebrafish forebrain. Neuron 28, 399–409. doi: 10.1016/S0896-6273(00)00120-3, PMID: 11144351

[ref27] Concha M. L. Russell C. Regan J. C. Tawk M. Sidi S. Gilmour D. T.. (2003). Local tissue interactions across the dorsal midline of the forebrain establish CNS laterality. Neuron 39, 423–438. doi: 10.1016/S0896-6273(03)00437-9, PMID: 12895418

[ref28] Concha M. L. Wilson S. W. (2001). Asymmetry in the epithalamus of vertebrates. J. Anatomy 199, 63–84. doi: 10.1046/j.1469-7580.2001.19910063.x, PMID: 11523830 PMC1594988

[ref29] Corballis M. C. (1997). The genetics and evolution of handedness. Psychol. Rev. 104, 714–727. doi: 10.1037/0033-295X.104.4.714, PMID: 9337630

[ref30] Costalunga G. Kobylkov D. Rosa-Salva O. Morandi-Raikova A. Vallortigara G. Mayer U. (2024). Responses in the left and right entopallium are differently affected by light stimulation in embryo. iScience 27:109268. doi: 10.1016/j.isci.2024.109268, PMID: 38439979 PMC10910295

[ref31] Dadda M. Domenichini A. Piffer L. Argenton F. Bisazza A. (2010a). Early differences in epithalamic left–right asymmetry influence lateralization and personality of adult zebrafish. Behav. Brain Res. 206, 208–215. doi: 10.1016/j.bbr.2009.09.019, PMID: 19765616

[ref32] Dadda M. Koolhaas W. H. Domenici P. (2010b). Behavioural asymmetry affects escape performance in a teleost fish. Biol. Lett. 6, 414–417. doi: 10.1098/rsbl.2009.0904, PMID: 20089537 PMC2880054

[ref33] de Carvalho T. N. Subedi A. Rock J. Harfe B. D. Thisse C. Thisse B.. (2014). Neurotransmitter map of the asymmetric dorsal habenular nuclei of zebrafish. Genesis 52, 636–655. doi: 10.1002/dvg.22785, PMID: 24753112 PMC4069259

[ref34] Deguchi T. Itoh M. Urawa H. Matsumoto T. Nakayama S. Kawasaki T.. (2009). Infrared laser-mediated local gene induction in medaka, zebrafish and *Arabidopsis thaliana*. Develop. Growth Differ. 51, 769–775. doi: 10.1111/j.1440-169X.2009.01135.x, PMID: 19843153

[ref35] Denenberg V. H. (1981). Hemispheric laterality in animals and the effects of early experience. Behav. Brain Sci. 4, 1–21. doi: 10.1017/S0140525X00007330

[ref36] Denenberg V. H. Garbanati J. Sherman G. Yutzey D. A. Kaplan R. (1978). Infantile stimulation induces brain lateralization in rats. Science 201, 1150–1152. doi: 10.1126/science.684436, PMID: 684436

[ref37] Draper B. W. Morcos P. A. Kimmel C. B. (2001). Inhibition of zebrafish fgf8 pre-mRNA splicing with morpholino oligos: a quantifiable method for gene knockdown. Genesis 30, 154–156. doi: 10.1002/gene.1053, PMID: 11477696

[ref38] Dreosti E. Llopis N. V. Carl M. Yaksi E. Wilson S. W. (2014). Left-right asymmetry is required for the habenulae to respond to both visual and olfactory stimuli. Curr. Biol. 24, 440–445. doi: 10.1016/j.cub.2014.01.016, PMID: 24508167 PMC3969106

[ref39] Duboc V. Dufourcq P. Blader P. Roussigné M. (2015). Asymmetry of the brain: development and implications. Annu. Rev. Genet. 49, 647–672. doi: 10.1146/annurev-genet-112414-055322, PMID: 26442849

[ref40] Duboué E. R. Hong E. Eldred K. C. Halpern M. E. (2017). Left Habenular Activity Attenuates Fear Responses in Larval Zebrafish. Current Biology. 27, 2154–2162. doi: 10.1016/j.cub.2017.06.017 28712566 PMC5570455

[ref41] Dundas E. Gastgeb H. Strauss M. S. (2012). Left visual field biases when infants process faces: a comparison of infants at high-and low-risk for autism spectrum disorder. J. Autism Dev. Disord. 42, 2659–2668. doi: 10.1007/s10803-012-1523-y, PMID: 22527700 PMC3408549

[ref42] Erter C. E. Solnica-Krezel L. Wright C. V. (1998). Zebrafish nodal-related 2Encodes an early mesendodermal inducer signaling from the extraembryonic yolk syncytial layer. Dev. Biol. 204, 361–372. doi: 10.1006/dbio.1998.9097, PMID: 9882476

[ref43] Essner R. A. Smith A. G. Jamnik A. A. Ryba A. R. Trutner Z. D. Carter M. E. (2017). AgRP neurons can increase food intake during conditions of appetite suppression and inhibit anorexigenic parabrachial neurons. J. Neurosci. 37, 8678–8687. doi: 10.1523/JNEUROSCI.0798-17.2017, PMID: 28821663 PMC5588461

[ref44] Facchin L. Burgess H. A. Siddiqi M. Granato M. Halpern M. E. (2009). Determining the function of zebrafish epithalamic asymmetry. Philos. Trans. Royal Society B: Biolog. Sci. 364, 1021–1032. doi: 10.1098/rstb.2008.0234, PMID: 19064346 PMC2666080

[ref45] Facchin L. Duboué E. R. Halpern M. E. (2015). Disruption of epithalamic left–right asymmetry increases anxiety in zebrafish. J. Neurosci. 35, 15847–15859. doi: 10.1523/JNEUROSCI.2593-15.2015, PMID: 26631467 PMC4666913

[ref46] Forrester G. S. Todd B. K. (2018). A comparative perspective on lateral biases and social behaviour. Prog. Brain Res. 238, 377–403. doi: 10.1016/bs.pbr.2018.06.014, PMID: 30097201

[ref47] Frasnelli E. Vallortigara G. Rogers L. J. (2012). Left-right asymmetries of behaviour and nervous system in invertebrates. Neurosci. Biobehav. Rev. 36, 1273–1291. doi: 10.1016/j.neubiorev.2012.02.006, PMID: 22353424

[ref48] Gamse J. T. Kuan Y. S. Macurak M. Brösamle C. Thisse B. Thisse C.. (2005). Directional asymmetry of the zebrafish epithalamus guides dorsoventral innervation of the midbrain target, Development. 132, 4869–4881. doi: 10.1242/dev.02046 16207761

[ref49] Gamse J. T. Shen Y. C. Thisse C. Thisse B. Raymond P. A. Halpern M. E.. (2002). Otx5 regulates genes that show circadian expression in the zebrafish pineal complex. Nat. Genet. 30, 117–121. doi: 10.1038/ng793, PMID: 11753388

[ref50] Gamse J. T. Thisse C. Thisse B. Halpern M. E. (2003). The parapineal mediates left-right asymmetry in the zebrafish diencephalon. Development 130, 1059–1068. doi: 10.1242/dev.00270, PMID: 12571098

[ref51] Ghirlanda S. Frasnelli E. Vallortigara G. (2009). Intraspecific competition and coordination in the evolution of lateralization. Philos. Trans. R. Soc. Lond. B 364, 861–866. doi: 10.1098/rstb.2008.0227, PMID: 19064359 PMC2666077

[ref52] Ghirlanda S. Vallortigara G. (2004). The evolution of brain lateralization: a game-theoretical analysis of population structure. Proc. Biol. Sci. 271, 853–857. doi: 10.1098/rspb.2003.2669, PMID: 15255105 PMC1691668

[ref53] Gostic M. Martinelli A. Tucker C. Yang Z. Gasparoli F. Ewart J. Y.. (2019). The dyslexia susceptibility KIAA0319 gene show a specific expression pattern during zebrafish development supporting a role beyond neural migration. J. Comp. Neurol. 527, 2634–2643. doi: 10.1002/cne.24696, PMID: 30950042 PMC6767054

[ref54] Gourronc F. Ahmad N. Nedza N. Eggleston T. Rebagliati M. (2007). Nodal activity around Kupffer's vesicle depends on the T-box transcription factors notail and spadetail and on notch signaling. Develop. Dynamics: Official Pub. American Association of Anatomists 236, 2131–2146. doi: 10.1002/dvdy.21249, PMID: 17654709

[ref55] Guglielmi L. Bühler A. Moro E. Argenton F. Poggi L. Carl M. (2020). Temporal control of Wnt signaling is required for habenular neuron diversity and brain asymmetry. Development 147:dev182865. doi: 10.1242/dev.182865, PMID: 32179574

[ref56] Güntürkün O. Diekamp B. Manns M. Nottelmann F. Prior H. Schwarz A.. (2000). Asymmetry pays: visual lateralization improves discrimination success in pigeons. Curr. Biol. 10, 1079–1081. doi: 10.1016/S0960-9822(00)00671-0, PMID: 10996079

[ref57] Güntürkün O. Kesch S. (1987). Visual lateralization during feeding in pigeons. Behav. Neurosci. 101, 433–435. doi: 10.1037/0735-7044.101.3.433, PMID: 3606815

[ref58] Güntürkün O. Ocklenburg S. (2017). Ontogenesis of lateralization. Neuron 94, 249–263. doi: 10.1016/j.neuron.2017.02.045, PMID: 28426959

[ref59] Güntürkün O. Ströckens F. Ocklenburg S. (2020). Brain lateralization: a comparative perspective. Physiol. Rev. 100, 1019–1063. doi: 10.1152/physrev.00006.2019 32233912

[ref60] Hashimoto Y. Maegawa S. Nagai T. Yamaha E. Suzuki H. Yasuda K.. (2004). Localized maternal factors are required for zebrafish germ cell formation. Dev. Biol. 268, 152–161. doi: 10.1016/j.ydbio.2003.12.013, PMID: 15031112

[ref61] Heuts B. A. (1999). Lateralization of trunk muscle volume, and lateralization of swimming turns of fish responding to external stimuli. Behav. Process. 47, 113–124. doi: 10.1016/S0376-6357(99)00056-X, PMID: 24896934

[ref62] Hojo M. Takashima S. Kobayashi D. Sumeragi A. Shimada A. Tsukahara T.. (2007). Right-elevated expression of charon is regulated by fluid flow in medaka Kupffer's vesicle. Develop. Growth Differ. 49, 395–405. doi: 10.1111/j.1440-169X.2007.00937.x, PMID: 17547649

[ref63] Hori M. (1993). Frequency-dependent natural selection in the handedness of scale-eating cichlid fish. Science 260, 216–219. doi: 10.1126/science.260.5105.216, PMID: 17807183

[ref64] Hüsken U. Carl M. (2013). The Wnt/beta-catenin signaling pathway establishes neuroanatomical asymmetries and their laterality. Mech. Dev. 130, 330–335. doi: 10.1016/j.mod.2012.09.002, PMID: 23022991

[ref65] Hüsken U. Stickney H. L. Gestri G. Bianco I. H. Faro A. Young R. M.. (2014). Tcf 7l2 is required for left-right asymmetric differentiation of habenular neurons. Curr. Biol. 24, 2217–2227. doi: 10.1016/j.cub.2014.08.006, PMID: 25201686 PMC4194317

[ref66] Hynd G. W. Semrud-Clikeman M. Lorys A. R. Novey E. S. Eliopulos D. (1990). Brain morphology in developmental dyslexia and attention deficit disorder/hyperactivity. Arch. Neurol. 47, 919–926. doi: 10.1001/archneur.1990.00530080107018, PMID: 2375699

[ref67] Inbal A. Kim S. H. Shin J. Solnica-Krezel L. (2007). Six3 represses nodal activity to establish early brain asymmetry in zebrafish. Neuron 55, 407–415. doi: 10.1016/j.neuron.2007.06.037, PMID: 17678854 PMC2032012

[ref68] Keehn B. Vogel-Farley V. Tager-Flusberg H. Nelson C. A. (2015). Atypical hemispheric specialization for faces in infants at risk for autism spectrum disorder. Autism Res. 8, 187–198. doi: 10.1002/aur.1438, PMID: 25808162 PMC4412772

[ref69] Keller P. J. Ahrens M. B. (2015). Visualizing whole-brain activity and development at the single-cell level using light-sheet microscopy. Neuron 85, 462–483. doi: 10.1016/j.neuron.2014.12.039, PMID: 25654253

[ref70] Kjelgaard M. M. Tager-Flusberg H. (2001). An investigation of language impairment in autism: implications for genetic subgroups. Lang. Cogn. Process. 16, 287–308. doi: 10.1080/01690960042000058, PMID: 16703115 PMC1460015

[ref71] Kohashi T. Oda Y. (2008). Initiation of Mauthner-or non-Mauthner-mediated fast escape evoked by different modes of sensory input. J. Neurosci. 28, 10641–10653. doi: 10.1523/JNEUROSCI.1435-08.2008, PMID: 18923040 PMC6671347

[ref72] Krishnan S. Mathuru A. S. Kibat C. Rahman M. Lupton C. E. Stewart J.. (2014). The right dorsal habenula limits attraction to an odor in zebrafish. Curr. Biol. 24, 1167–1175. doi: 10.1016/j.cub.2014.03.073, PMID: 24856207

[ref73] Kuan Y. S. Gamse J. T. Schreiber A. M. Halpern M. E. (2007). Selective asymmetry in a conserved forebrain to midbrain projection. J. Exp. Zool. B Mol. Dev. Evol. 308, 669–678. doi: 10.1002/jez.b.21184, PMID: 17592620

[ref74] Lagutin O. V. Zhu C. C. Kobayashi D. Topczewski J. Shimamura K. Puelles L.. (2003). Six3 repression of Wnt signaling in the anterior neuroectoderm is essential for vertebrate forebrain development. Genes Dev. 17, 368–379. doi: 10.1101/gad.1059403, PMID: 12569128 PMC195989

[ref75] Lekk I. Duboc V. Faro A. Nicolaou S. Blader P. Wilson S. W. (2019). Sox 1a mediates the ability of the parapineal to impart habenular left-right asymmetry. eLife 8:e47376. doi: 10.7554/eLife.47376, PMID: 31373552 PMC6677535

[ref76] Liang J. O. Etheridge A. Hantsoo L. Rubinstein A. L. Nowak S. J. Izpisúa Belmonte J. C. (2000). Asymmetric nodal signaling in the zebrafish diencephalon positions the pineal organ. Development 127, 5101–5112. doi: 10.1242/dev.127.23.5101, PMID: 11060236

[ref77] Lombardo M. V. Pierce K. Eyler L. T. Barnes C. C. Ahrens-Barbeau C. Solso S.. (2015). Different functional neural substrates for good and poor language outcome in autism. Neuron 86, 567–577. doi: 10.1016/j.neuron.2015.03.023, PMID: 25864635 PMC4610713

[ref78] Long S. Ahmad N. Rebagliati M. (2003). The zebrafish nodal-related gene southpaw is required for visceral and diencephalic left-right asymmetry. Development 130, 2303–2316. doi: 10.1242/dev.00436, PMID: 12702646

[ref79] Mac Neilage P. F. Rogers L. J. Vallortigara G. (2009). Origins of the left and right brain. Sci. Am. 301, 60–67. doi: 10.1038/scientificamerican0709-60, PMID: 19555025

[ref80] Masulli P. Galazka M. Eberhard D. Johnels J. Å. Gillberg C. Billstedt E.. (2022). Data-driven analysis of gaze patterns in face perception: methodological and clinical contributions. Cortex 147, 9–23. doi: 10.1016/j.cortex.2021.11.011, PMID: 34998084

[ref81] Maynard Smith J. (1982). Evolution and the theory of the game. Cambridge: Cambridge University Press.

[ref82] McCleery J. P. Akshoomoff N. Dobkins K. R. Carver L. J. (2009). Atypical face versus object processing and hemispheric asymmetries in 10-month-old infants at risk for autism. Biol. Psychiatry 66, 950–957. doi: 10.1016/j.biopsych.2009.07.031, PMID: 19765688 PMC2783702

[ref83] McManus I. C. (1999). “Handedness, cerebral lateralization, and the evolution of language” in The descent of mind: Psychological perspectives on hominid evolution. eds. Corballis M. C. Lea S. E. G. (Oxford: Oxford University Press), 194–217.

[ref84] Melby A. E. Warga R. M. Kimmel C. B. (1996). Specification of cell fates at the dorsal margin of the zebrafish gastrula. Development 122, 2225–2237. doi: 10.1242/dev.122.7.2225, PMID: 8681803

[ref85] Mervis C. B. Morris C. A. Bertrand J. Robinson B. F. (1999). Williams syndrome: Findings from an integrated program of research. In Neurodevelopmental disorders. ed. Tager-Flusberg H. (The MIT Press), pp. 65–110.

[ref86] Messina A. Boiti A. Vallortigara G. (2021). Asymmetric distribution of pallial-expressed genes in zebrafish (*Danio rerio*). Eur. J. Neurosci. 53, 362–375. doi: 10.1111/ejn.14914, PMID: 32692463

[ref87] Messina A. Sovrano V. A. Baratti G. Musa A. Gobbo A. Adiletta A.. (2024). Valproic acid exposure affects social visual lateralization and asymmetric gene expression in zebrafish larvae. Sci. Rep. 14:4474. doi: 10.1038/s41598-024-54356-7, PMID: 38395997 PMC10891151

[ref88] Miklosi A. Andrew R. J. (1999). Right eye use associated with decision to bite in zebrafish. Behav. Brain Res. 105, 199–205. doi: 10.1016/S0166-4328(99)00071-6, PMID: 10563493

[ref89] Miklosi A. Andrew R. J. Savage H. (1997). Behavioural lateralisation of the tetrapod type in the zebrafish (*brachydanio rerio*). Physiol. Behav. 63, 127–135. doi: 10.1016/S0031-9384(97)00418-6, PMID: 9402625

[ref90] Miletto Petrazzini M. E. Sovrano V. A. Vallortigara G. Messina A. (2020). Brain and behavioral asymmetry: a lesson from fish. Front. Neuroanat. 14:11. doi: 10.3389/fnana.2020.00011, PMID: 32273841 PMC7113390

[ref91] Miyasaka N. Morimoto K. Tsubokawa T. Higashijima S. I. Okamoto H. Yoshihara Y. (2009). From the olfactory bulb to higher brain centers: genetic visualization of secondary olfactory pathways in zebrafish. J. Neurosci. 29, 4756–4767. doi: 10.1523/JNEUROSCI.0118-09.2009, PMID: 19369545 PMC6665349

[ref93] Neugebauer J. M. Yost H. J. (2014). FGF signaling is required for brain left–right asymmetry and brain midline formation. Dev. Biol. 386, 123–134. doi: 10.1016/j.ydbio.2013.11.020, PMID: 24333178 PMC3970204

[ref94] Newman M. Ebrahimie E. Lardelli M. (2014). Using zebrafish model for Alzheimer’s disease research. Front. Genet. 5, 1–10. doi: 10.3389/fgene.2014.00189, PMID: 25071820 PMC4075077

[ref95] Nottebohm F. (1971). Neural lateralization of vocal control in a passerine bird. I. Song. J. Exp. Zool. 177, 229–261. doi: 10.1002/jez.1401770210, PMID: 5571594

[ref96] Ocklenburg S. Ströckens F. Güntürkün O. (2013). Lateralisation of conspecific vocalisation in non-human vertebrates. Laterality 18, 1–31. doi: 10.1080/1357650X.2011.626561, PMID: 23231542

[ref97] Pandey S. Shekhar K. Regev A. Schier A. F. (2018). Comprehensive identification and spatial mapping of habenular neuronal types using single-cell RNA-seq. Curr. Biol. 28, 1052–1065.e7. doi: 10.1016/j.cub.2018.02.040, PMID: 29576475 PMC6042852

[ref98] Park J. W. Choi T. I. Kim T. Y. Lee Y. R. Don D. W. George-Abraham J. K.. (2024). RFC2 may contribute to the pathogenicity of Williams syndrome revealed in a zebrafish model. J. Genetics & Genomics 51, 1389–1403. doi: 10.1016/j.jgg.2024.09.016, PMID: 39368701 PMC11624490

[ref99] Pober B. R. (2010). williams–Beuren syndrome. N. Engl. J. Med. 362, 239–252. doi: 10.1056/NEJMra0903074, PMID: 20089974

[ref100] Powell G. T. Faro A. Zhao Y. Stickney H. Novellasdemunt L. Henriques P.. (2024). Cachd1 interacts with Wnt receptors and regulates neuronal asymmetry in the zebrafish brain. Science 384, 573–579. doi: 10.1126/science.ade6970, PMID: 38696577 PMC7615972

[ref101] Raya A. Koth C. M. Büscher D. Kawakami Y. Itoh T. Raya R. M.. (2003). Activation of notch signaling pathway precedes heart regeneration in zebrafish. Proc. Natl. Acad. Sci. 100, 11889–11895. doi: 10.1073/pnas.1834204100 12909711 PMC304103

[ref102] Rebagliati M. R. Toyama R. Fricke C. Haffter P. Dawid I. B. (1998). Zebrafish nodal-related genes are implicated in axial patterning and establishing left–right asymmetry. Dev. Biol. 199, 261–272. doi: 10.1006/dbio.1998.8935, PMID: 9698446

[ref103] Regan J. C. Concha M. L. Roussigne M. Russell C. Wilson S. W. (2009). An Fgf 8-dependent bistable cell migratory event establishes CNS asymmetry. Neuron 61, 27–34. doi: 10.1016/j.neuron.2008.11.030, PMID: 19146810 PMC2790412

[ref104] Reifers F. Böhli H. Walsh E. C. Crossley P. H. Stainier D. Y. Brand M. (1998). Fgf8 is mutated in zebrafish acerebellar (ace) mutants and is required for maintenance of midbrain-hindbrain boundary development and somitogenesisy. Development 125, 2381–2395. doi: 10.1242/dev.125.13.2381, PMID: 9609821

[ref105] Ribasés M. Bosch R. Hervás A. Ramos-Quiroga J. A. Sánchez-Mora C. Bielsa A.. (2009). Case-control study of six genes asymmetrically expressed in the two cerebral hemispheres: association of BAIAP2 with attention-deficit/hyperactivity disorder. Biol. Psychiatry 66, 926–934. doi: 10.1016/j.biopsych.2009.06.024, PMID: 19733838

[ref106] Rogers L. J. Anson J. M. (1979). Lateralization of function in chicken forebrain. Pharmacol. Biochem. Behav. 10, 679–686. doi: 10.1016/0091-3057(79)90320-4, PMID: 493285

[ref107] Rogers L. J. Vallortigara G. (2017). Lateralized brain functions. New York: Springer.

[ref108] Rogers L. J. Vallortigara G. Andrew R. J. (2013). Divided brains: The biology and behaviour of brain asymmetries. Cambridge: Cambridge University Press. 5, 1–10.

[ref109] Rogers L. J. Zucca P. Vallortigara G. (2004). Advantages of having a lateralized brain. Proc. R. Soc. B 271, S420–S422. doi: 10.1098/rsbl.2004.0200, PMID: 15801592 PMC1810119

[ref110] Roussigné M. Bianco I. H. Wilson S. W. Blader P. (2009). Nodal signalling imposes left-right asymmetry upon neurogenesis in the habenular nuclei. Development 136, 1549–1557. doi: 10.1242/dev.034793, PMID: 19363156 PMC2675782

[ref111] Roussigne M. Blader P. Wilson S. W. (2012). Breaking symmetry: the zebrafish as a model for understanding left-right asymmetry in the developing brain. Dev. Neurobiol. 72, 269–281. doi: 10.1002/dneu.20885, PMID: 22553774

[ref112] Sagasti A. (2007). Three ways to make two sides: genetic models of asymmetric nervous system development. Neuron 55, 345–351. doi: 10.1016/j.neuron.2007.07.015, PMID: 17678849

[ref113] Samara A. Vougas K. Papadopoulou A. Anastasiadou E. Baloyanni N. Paronis E.. (2011). Proteomics reveal rat hippocampal lateral asymmetry. Hippocampus 21, 108–119. doi: 10.1002/hipo.20727, PMID: 20020437

[ref114] Sampath K. Rubinstein A. L. Cheng A. M. Liang J. O. Fekany K. Solnica-Krezel L.. (1998). Induction of the zebrafish ventral brain and floorplate requires cyclops/nodal signalling. Nature 395, 185–189. doi: 10.1038/26020, PMID: 9744278

[ref115] Satou C. Kimura Y. Kohashi T. Horikawa K. Takeda H. Oda Y.. (2009). Functional role of a specialized class of spinal commissural inhibitory neurons during fast escapes in zebrafish. J. Neurosci. 29, 6780–6793. doi: 10.1523/JNEUROSCI.0801-09.2009, PMID: 19474306 PMC6665578

[ref116] Shalev R. S. Manor O. Amir N. Wertman-Elad R. Gross-Tsur V. (1995). Developmental dyscalculia and brain laterality. Cortex 31, 357–365. doi: 10.1016/S0010-9452(13)80368-1, PMID: 7555012

[ref117] Snelson C. D. Burkart J. T. Gamse J. T. (2008). Formation of the asymmetric pineal complex in zebrafish requires two independently acting transcription factors. Develop. Dynamics: Official Pub. American Association of Anatomists 237, 3538–3544. doi: 10.1002/dvdy.21607, PMID: 18629869 PMC2810829

[ref118] Snelson C. D. Gamse J. T. (2009). Building an asymmetric brain: development of the zebrafish epithalamus. Semin. Cell Dev. Biol. 20, 491–497. doi: 10.1016/j.semcdb.2008.11.008, PMID: 19084075 PMC2729063

[ref119] Sovrano V. A. (2004). Visual lateralization in response to familiar and unfamiliar stimuli in fish. Behav. Brain Res. 152, 385–391. doi: 10.1016/j.bbr.2003.10.022, PMID: 15196807

[ref120] Sovrano V. A. Andrew R. J. (2006). Eye use during viewing a reflection: behavioural lateralisation in zebrafish larvae. Behav. Brain Res. 167, 226–231. doi: 10.1016/j.bbr.2005.09.021, PMID: 16298436

[ref121] Sovrano V. A. Bisazza A. Vallortigara G. (2001). Lateralization of response to social stimuli in fishes: a comparison between different methods and species. Physiol. Behav. 74, 237–244. doi: 10.1016/S0031-9384(01)00552-2, PMID: 11564473

[ref122] Sovrano V. A. Rainoldi C. Bisazza A. Vallortigara G. (1999). Roots of brain specializations: preferential left-eye use during mirror-image inspection in six species of teleost fish. Behav. Brain Res. 106, 175–180. doi: 10.1016/S0166-4328(99)00105-9, PMID: 10595433

[ref123] Stancher G. Sovrano V. A. Vallortigara G. (2018). Motor asymmetries in fishes, amphibians, and reptiles. Prog. Brain Res. 238, 33–56. doi: 10.1016/bs.pbr.2018.06.002, PMID: 30097199

[ref124] Ströckens F. Güntürkün O. Ocklenburg S. (2013). Limb preferences in non-human vertebrates. Laterality 18, 536–575. doi: 10.1080/1357650X.2012.723008, PMID: 23167450

[ref125] Sun T. Patoine C. Abu-Khalil A. Visvader J. Sum E. Cherry T. J.. (2005). Early asymmetry of gene transcription between embryonic human left and right cerebral cortex. Science 308, 1794–1798. doi: 10.1126/science.1110324, PMID: 15894532 PMC2756725

[ref126] Takeuchi M. Kaneko H. Nishikawa K. Kawakami K. Yamamoto M. Kobayashi M. (2010). Efficient transient rescue of hematopoietic mutant phenotypes in zebrafish using Tol 2-mediated transgenesis. Develop. Growth Differ. 52, 245–250. doi: 10.1111/j.1440-169X.2009.01168.x, PMID: 20100247

[ref127] Thompson P. M. Hayashi K. M. De Zubicaray G. Janke A. L. Rose S. E. Semple J.. (2003). Dynamics of gray matter loss in Alzheimer's disease. J. Neurosci. 23, 994–1005. doi: 10.1523/JNEUROSCI.23-03-00994.2003, PMID: 12574429 PMC6741905

[ref128] Toga A. W. Thompson P. M. (2003). Mapping brain asymmetry. Nat. Rev. Neurosci. 4, 37–48. doi: 10.1038/nrn1009, PMID: 12511860

[ref129] Torres-Perez J. Anagianni S. Mech A. M. Havelange W. Garcia-Gonzalez J. Fraser S. E.. (2023). Baz 1b loss-of-function in zebrafish produces phenotypic alterations consistent with the domestication syndrome. iScience 26:105704. doi: 10.1016/j.isci.2022.105704, PMID: 36582821 PMC9793288

[ref130] Torres-Perez J. Leggieri A. Mech A. M. Anagianni S. Havelange W. Brennan C. H. (2024). Disrupting fzd9b in zebrafish recapitulates stress-and anxiety-like behaviours associated with Williams syndrome. biorXiv. doi: 10.1101/2024.01.22.576596 PMC13503493 42637991

[ref131] Vallortigara G. (1992). Right hemisphere advantage for social recognition in the chick. Neuropsychologia 30, 761–768. doi: 10.1016/0028-3932(92)90080-6, PMID: 1407491

[ref9001] Vallortigara G. (2000). Comparative neuropsychology of the dual brain: a stroll through animals’ left and right perceptual worlds. Brain and language 73, 189–219. doi: 10.1006/brln.2000.2303 10856174

[ref132] Vallortigara G. (2006). The evolutionary psychology of left and right: costs and benefits of lateralization. Dev. Psychobiol. 48, 418–427. doi: 10.1002/dev.20166, PMID: 16886183

[ref133] Vallortigara G. Bisazza A. (2002). “How ancient is brain lateralization?” in Comparative Vertebrate Lateralization. eds. Andrew R. J. Rogers L. J. (Cambridge: Cambridge University Press), 9–69.

[ref134] Vallortigara G. Chiandetti C. Sovrano V. A. (2011). Brain asymmetry (animal). Wiley Interdiscip. Rev. Cogn. Sci. 2, 146–157. doi: 10.1002/wcs.100, PMID: 26302006

[ref135] Vallortigara G. Rogers L. (2005). Survival with an asymmetrical brain: advantages and disadvantages of cerebral lateralization. Behav. Brain Sci. 28, 575–589. doi: 10.1017/S0140525X05000105, PMID: 16209828

[ref136] Vallortigara G. Rogers L. J. (2020). A function for the bicameral mind. Cortex 124, 274–285. doi: 10.1016/j.cortex.2019.11.018, PMID: 32058074

[ref137] Vallortigara G. Versace E. (2017). Laterality at the neural, cognitive, and behavioral levels. In “APA handbook of comparative psychology: Vol. 1. Basic concepts, methods, neural substrate, and behavior”, Call J. (editor-in-chief), pp. 557–577, American Psychological Association, Washington DC.

[ref138] Vallortigara G. Vitiello G. (2024). Brain asymmetry as minimization of free energy: a theoretical model. R. Soc. Open Sci. 11:240465. doi: 10.1098/rsos.240465, PMID: 39086831 PMC11289647

[ref139] Versace E. Sgadò P. George G. Loveland J. L. Ward J. Thorpe P.. (2022). Light-induced asymmetries in embryonic retinal gene expression are mediated by the vascular system and extracellular matrix. Sci. Rep. 12:12086. doi: 10.1038/s41598-022-14963-8, PMID: 35840576 PMC9287303

[ref140] Wang X. Zhang J. B. He K. J. Wang F. Liu C. F. (2021). Advances of zebrafish in neurodegenerative disease: from models to drug discovery. Front. Pharmacol. 12:713963. doi: 10.3389/fphar.2021.713963, PMID: 34335276 PMC8317260

[ref141] Watkins J. Miklósi A. Andrew R. J. (2004). Early asymmetries in the behaviour of zebrafish larvae. Behav. Brain Res. 151, 177–183. doi: 10.1016/j.bbr.2003.08.012, PMID: 15084433

